# Scrotal Sarcoidosis: A Case Report With Radiological Insights

**DOI:** 10.7759/cureus.71172

**Published:** 2024-10-09

**Authors:** Yehia Hassan, Eoin O'Doherty, Matthew J Rewhorn, Daniel Heffernan Ho, Ahmed H Gabr

**Affiliations:** 1 Urology, National Health Service (NHS) Greater Glasgow and Clyde, Glasgow, GBR; 2 Radiology, National Health Service (NHS) Lanarkshire, Glasgow, GBR; 3 Radiology, National Health Service (NHS) Greater Glasgow and Clyde, Glasgow, GBR; 4 Urology, Minia University, Minia, EGY

**Keywords:** case report, ct, granulomatous disorder, lymphadenopathy, mri, non-caseating granulomas, sarcoidosis, scrotal, testicular mass, ultrasound imaging

## Abstract

Sarcoidosis is an idiopathic inflammatory systemic disorder that is believed to be caused by a mixture of environmental factors and genetic predisposition. Less than 50 cases of testicular sarcoidosis have been presented in the literature over the last two decades, making it an extremely rare form of the disease. Furthermore, the radiological pathognomonic features are less well documented. We present a 58-year-old man who was referred with a two-week history of painless right-sided scrotal swelling with the radiological finding of scrotal sarcoidosis.

## Introduction

Sarcoidosis is an idiopathic inflammatory systemic disorder that is believed to be caused by a mixture of environmental factors and genetic predisposition. The histological hallmark of sarcoidosis is the presence of non-caseating granulomas [1], and Caeser Boeck first expressed the term sarcoid in 1899 due to the histological similarities to a sarcoma [2]. More than 90% of people with sarcoidosis experience respiratory involvement as their most frequent symptom, and genitourinary tract involvement is rare [3]. Less than 50 cases of testicular sarcoidosis have been presented in the literature over the last two decades, making it an extremely rare form of the disease, and furthermore, the radiological pathognomonic features are less well documented [4].

## Case presentation

We present a 58-year-old man who was referred with a two-week history of painless right-sided scrotal swelling. He denied urinary symptoms and had no relevant sexual history or extra urinary manifestations. His past medical history revealed urinary stones and essential hypertension. Clinical examination confirmed a right hydrocele with bilateral grade 2 varicocele.

**Figure 1:**
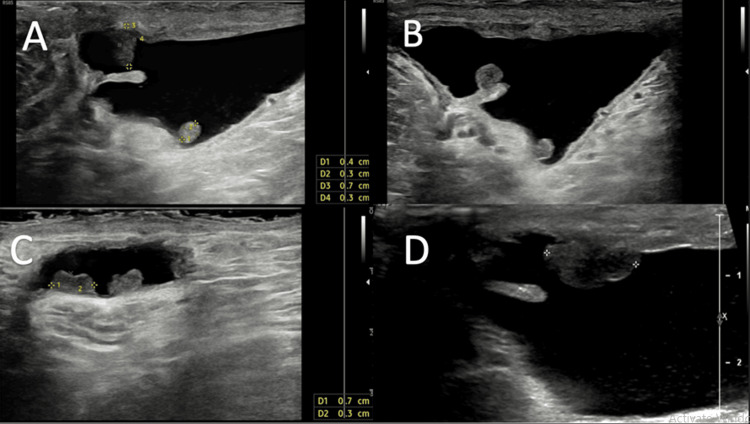
Longitudinal ultrasound images of the right scrotal wall (A-D) The right scrotal wall area. There is a small hydrocele showing multiple sub-centimeter nodules projecting into the hydrocele (A-C). The largest nodule (D) demonstrates reduced echogenicity.

To further investigate, a scrotal ultrasound was organized (Figure 1).

**Figure 2:**
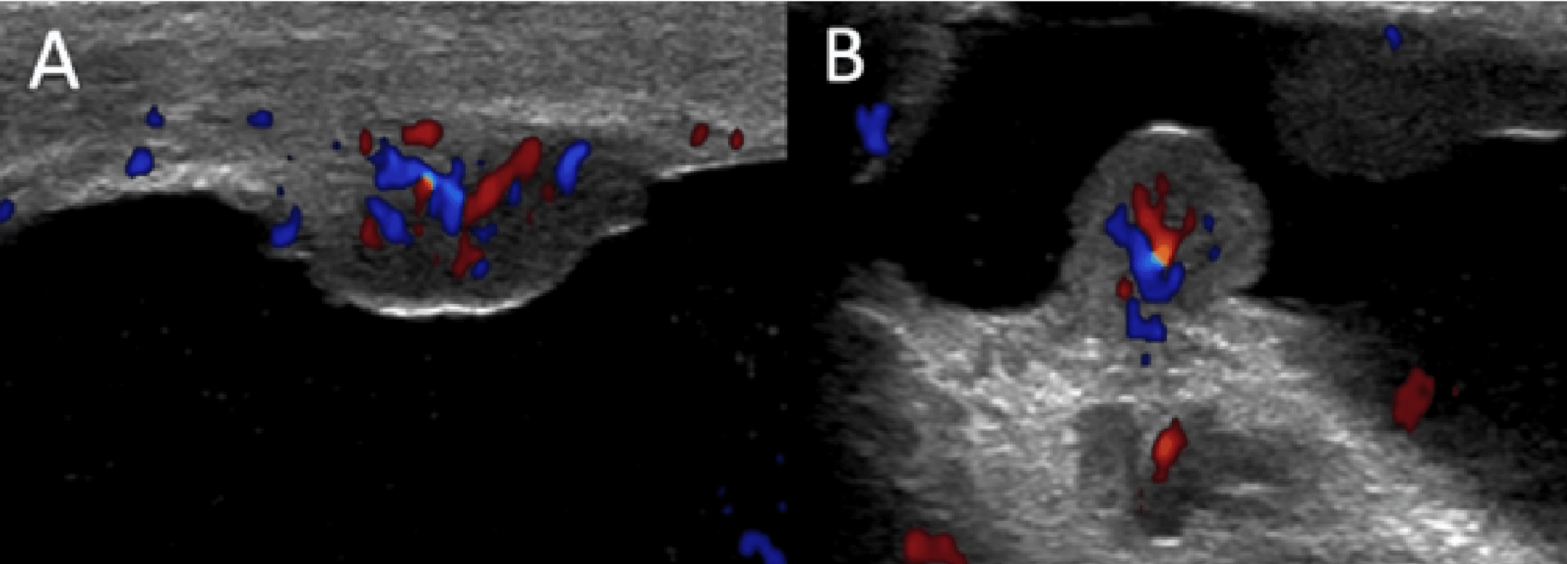
Doppler ultrasound of the right scrotal wall (A,B) Moderately increased vascularity of the soft-tissue nodules within the right scrotum.

Ultrasound scans (USS) confirmed the clinical findings of right-sided hydrocele and bilateral varicocele. However, the radiologist also commented on an unusual finding of multiple solid masses, which were highly vascular and projected into the hydrocele, with the largest measuring approximately 1 cm (Figure 2).

A renal ultrasound showed a 3.5 cm cyst in the lower pole of the left kidney, and a triple phase CT performed thereafter confirmed a Bosniak 2 cyst with no routine follow-up required.

**Figure 3:**
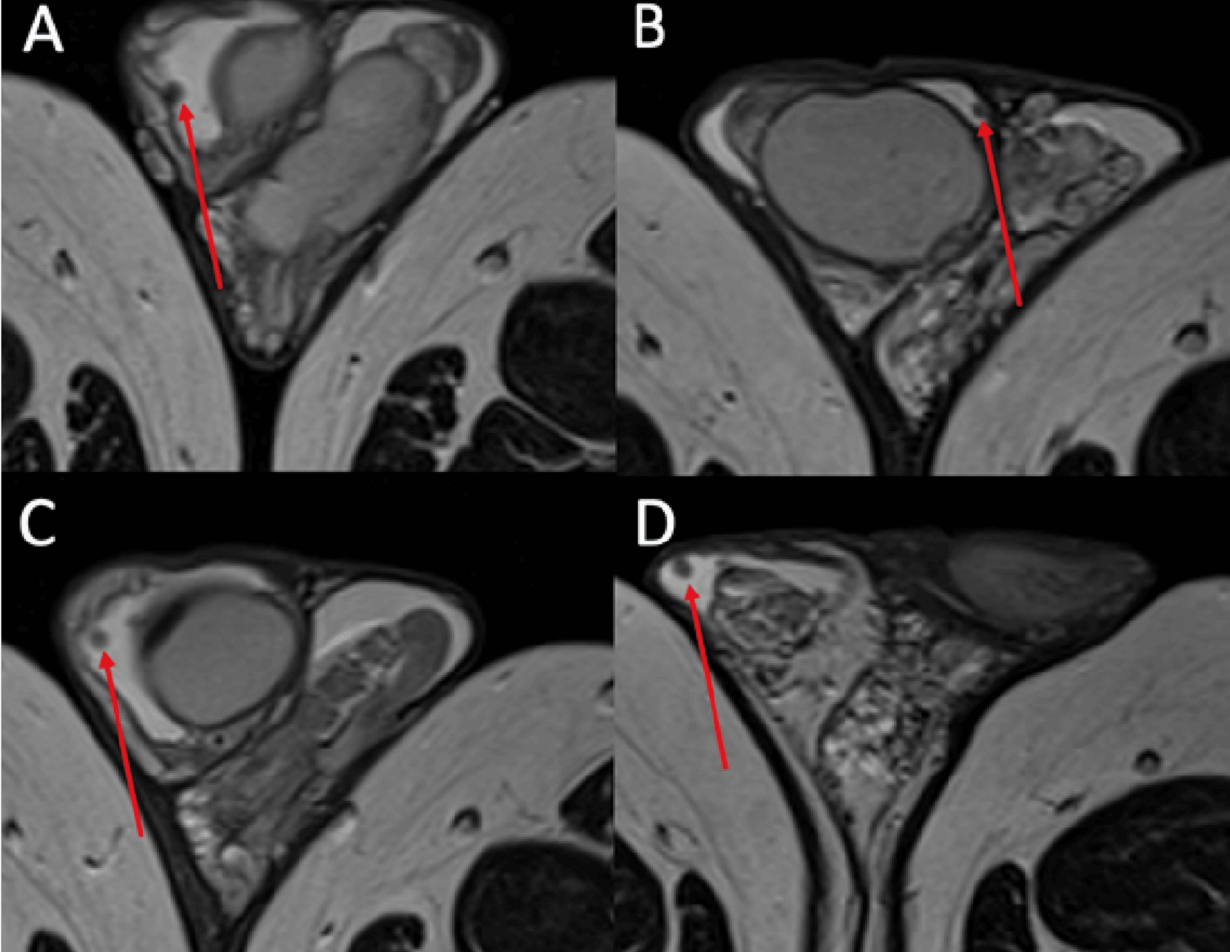
Axial T2-weighted MRI The red arrow points to nodules within the parietal layer of the right tunica vaginalis. These display T2-weighted low intensity in the lateral scrotal wall (A,C), medial wall (B), and anterior scrotum (D).

**Figure 4:**
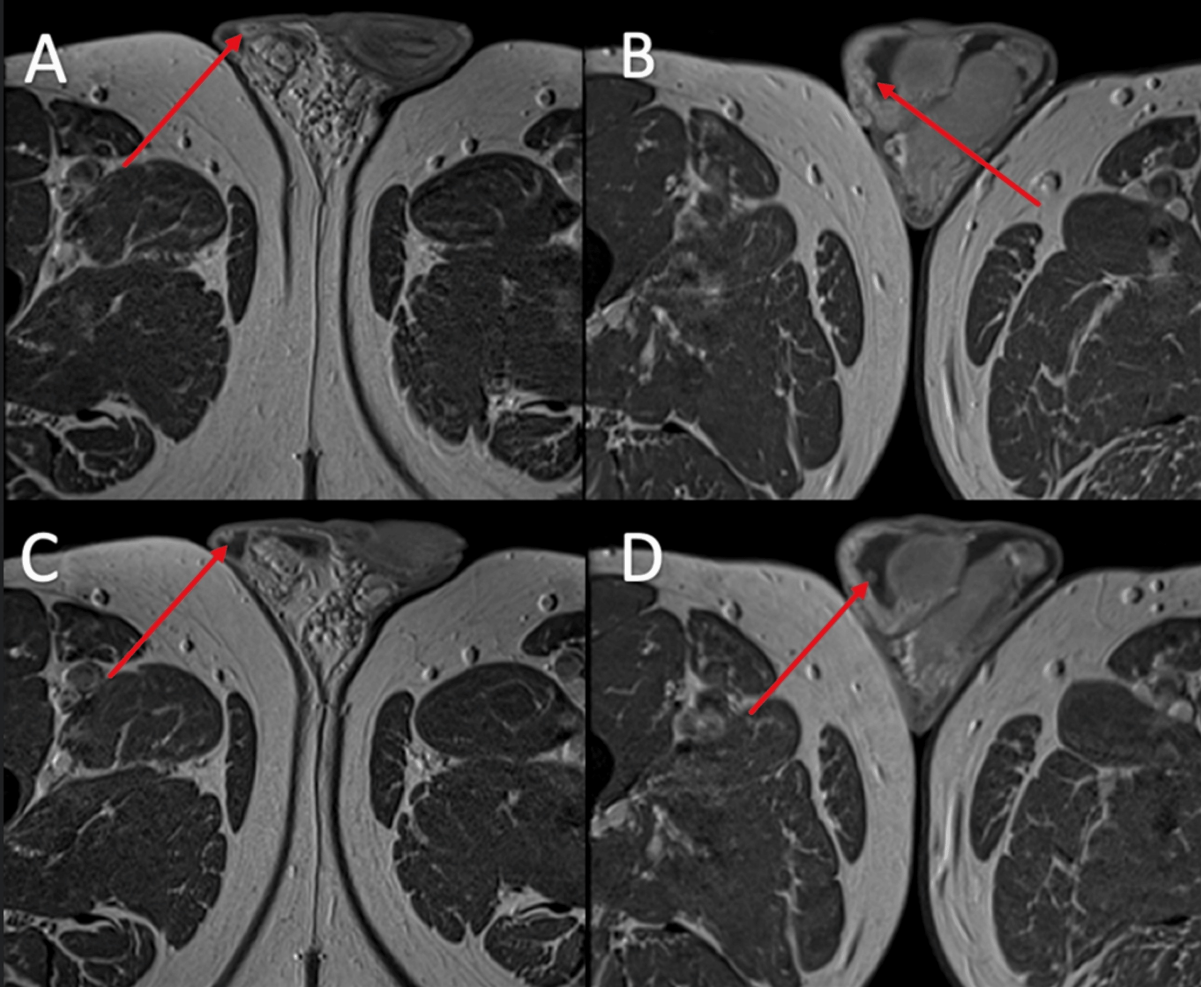
Post-gadolinium contrast T1-weighted axial MRI Peripheral contrast enhancement of nodules (A,C), with general enhancement of smaller nodules (B,D).

**Figure 5:**
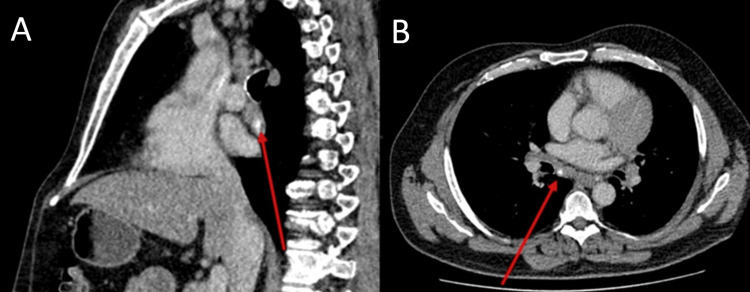
Contrast-enhanced CT thorax Sagittal (A) and axial (B) images demonstrating calcification within an enlarged subcarinal lymph node, highlighted by the red arrow.

The scrotal ultrasound images were reviewed in the departmental uroradiology meeting, and the radiologist suggested a three-monthly interval scan, which showed unchanged appearances. The radiologist raised the concern of a scrotal sarcoid and suggested an MRI of the scrotum (Figure 3 and Figure 4), in addition to a CT of the chest, abdomen, and pelvis (Figure 5).

The MRI showed that the nodularity described in the ultrasound report is confined to the tunica vaginalis and had reduced in size compared to the ultrasound. The report findings were consistent with a scrotal sarcoid. 

The CT showed enlarged bilateral hilar and mediastinal lymph nodes with a calcified subcarinal lymph node (Figure 5).

The patient did not receive any treatment and was reviewed by the respiratory team, who decided that no intervention was required since there were no respiratory symptoms. Follow-up would consist of interval CT scans, with the possibility of endobronchial ultrasound (EBUS) if the lymph nodes enlarge. The patient was not keen to proceed with a testicular biopsy, given the absence of symptoms.

The patient consented in handwriting to publish the figures from his scans.

## Discussion

Sarcoidosis is a granulomatous disorder that can affect different organs with respiratory involvement. Most patients classically present with bilateral hilar lymphadenopathy, parenchymal disease of the lung, and eye or skin lesions [5]; however, up to 70% of patients can develop extrathoracic manifestations, with the most commonly involved sites including the skin, lymph nodes, and eyes [6]. Genitourinary sarcoidosis is rare and reported in less than 0.2% of the clinically reported cases [7]. Using a combination of history, examination, blood tests, including full blood count, tumor markers, serum angiotensin-converting enzyme (ACE), QuantiFERON Gold, IgG4, ultrasound, and MRI can help differentiate between the rarer causes of unilateral or bilateral scrotal enlargement.

The most significant differential diagnosis of a scrotal mass is testicular malignancy; however, benign conditions, including hydrocele, epididymal cysts, varicocele, and inguinal hernia, can also impact a patient. As well as these common conditions, urologists need to be aware of rarer causes, including TB, para-testicular tumors, such as sarcoma, IgG4 disease, and sarcoidosis. To help differentiate between these pathologies, a combination of history, examination, blood tests, and imaging, namely testicular ultrasound, is utilized. [8]

The common radiological features of sarcoid are bilateral hilar lymphadenopathy, interstitial lung disease, calcification of lymph nodes, and, more rarely, pleural effusion and pleural thickening. 

In our report, sarcoid was initially thought of due to the radiological appearances being typical for a pseudotumor-type etiology, namely, multiple well-defined tunica-based, extra-testicular lesions, which show reduced echogenicity and relapsing and remitting [9]

However, it is not possible to make a diagnosis of scrotal sarcoid on ultrasound appearance alone. In our case, the MRI appearances also demonstrated the expected findings of sarcoid, which included low T2-weighted signal intensity (Figure 3) and enhancing nodules on T1-weighted contrast sequences (Figure 4). These features are not specific and can be seen in the fibrous pseudotumor of the scrotum (FPOS), which is the main differential. Given the patient's extra-testicular manifestations on CT, which demonstrated calcified lymphadenopathy, the diagnosis of scrotal sarcoid was thought more likely than fibrous pseudotumor. In addition to that, on ultrasound of FPOS, you would expect to see acoustic shadowing posterior to lesions due to the fibrous and calcified contents within the lesions [10]. This was not observed on ultrasound in our case, where the returning ultrasound signal posterior to the lesions was intact. Color Doppler of FPOS typically shows minimal vascularity, again owing to the fibrous nature of the contents [11], whereas in our case, the lesions were hyper-vascular.

Another rare differential diagnosis is testicular mesothelioma. The patient had an ultrasound 11 years ago that showed scrotal lesions of unknown etiology. Given that the 10-year survival rate for testicular mesothelioma is 33% [12], this diagnosis is unlikely in our patient, who has experienced no deterioration or symptoms.

Testicular lymphoma is another differential, which you would expect to display marked hyperemia or hypervascularity on Doppler ultrasound [13], typically more pronounced compared to sarcoidosis. However, it usually presents with classic constitutional symptoms, which were absent in our case. That said, CT provided clarity in distinguishing this differential, as calcified lymphadenopathy would not be expected unless the person had a previously treated lymphoma, which was not the case for our patient. In contrast, sarcoidosis is a key differential diagnosis for intrathoracic lymphadenopathy [14].

Another differential diagnosis is testicular sarcoma, though only a few cases have been reported in the literature. Its ultrasound findings can be comparable to scrotal sarcoidosis, but testicular sarcoma is described as more heterogeneous than what we found in this case. MRI findings were similar with regards to T1-weighted and T2-weighted imaging, but unlike our case, no restricted diffusion was shown [15]. In contrast, our case showed restricted diffusion on diffusion-weighted imaging (DWI) sequences. Also, the longstanding, relapsing, and remitting nature of this case also makes sarcoma an unlikely diagnosis.

## Conclusions

Sarcoidosis is considered a benign and self-limiting condition, with a rare manifestation being genitourinary sarcoidosis. This presentation can mimic other conditions, including malignancy, making an accurate diagnosis challenging. A combination of the clinical examination findings, blood tests, imaging results, and histological examination of a biopsy taken from the lesion would ideally establish the diagnosis. In cases where multiple atypical lesions are seen in the testicles by ultrasonography, sarcoidosis should be considered and investigated as a differential diagnosis, which may prevent patients from undergoing more invasive tests or biopsies. In such cases and in the absence of well-established guidelines, a multidisciplinary team approach is essential for monitoring and treatment.
